# Recruitment and retention of young women into nutrition research studies: practical considerations

**DOI:** 10.1186/1745-6215-15-23

**Published:** 2014-01-16

**Authors:** Alecia Leonard, Melinda Hutchesson, Amanda Patterson, Kerry Chalmers, Clare Collins

**Affiliations:** 1 Priority Research Centre in Physical Activity and Nutrition and School of Health Sciences, Faculty of Health and Medicine, University of Newcastle, University Drive, Callaghan, Newcastle NSW 2308, Australia; 2 School of Psychology, Faculty of Science and IT, University of Newcastle, NSW Newcastle, Australia

**Keywords:** Young women, Recruitment, Retention, Nutrition study

## Abstract

**Background:**

Successful recruitment and retention of participants into research studies is critical for optimising internal and external validity. Research into diet and lifestyle of young women is important due to the physiological transitions experienced at this life stage. This paper aims to evaluate data related to recruitment and retention across three research studies with young women, and present practical advice related to recruiting and retaining young women in order to optimise study quality within nutrition research.

**Methods:**

Recruitment and retention strategies used in three nutrition studies that targeted young women (18 to 35 years) were critiqued. A randomised controlled trial (RCT), a crossover validation study and a cross-sectional survey were conducted at the University of Newcastle, Australia between 2010 and 2013Successful recruitment was defined as maximum recruitment relative to time. Retention was assessed as maximum participants remaining enrolled at study completion.

**Results:**

Recruitment approaches included notice boards, web and social network sites (Facebook and Twitter), with social media most successful in recruitment. The online survey had the highest recruitment in the shortest time-frame (751 participants in one month). Email, phone and text message were used in study one (RCT) and study two (crossover validation) and assisted in low attrition rates, with 93% and 75.7% completing the RCT and crossover validation study respectively. Of those who did not complete the RCT, reported reasons were: being too busy; and having an unrelated illness.

**Conclusion:**

Recruiting young women into nutrition research is challenging. Use of social media enhances recruitment, while Email, phone and text message contact improves retention within interventions. Further research comparing strategies to optimise recruitment and retention in young women, including flexible testing times, reminders and incentives is warranted.

## Background

Attracting young women to participate in nutrition, health and medical research is essential in developing translatable diet, health and lifestyle education programs relevant to their life stage [1]. However, difficulties exist in recruiting young women and keeping them involved in research studies [2]. Very little research into factors affecting their recruitment and engagement in nutrition research has been conducted [3]. From the limited research conducted to date, some common challenges have been highlighted, such as making initial contact, arranging mutually suitable times for data collection, maintaining contact for study duration and reducing attrition [2,4,5]. These challenges appear to be less prevalent in the recruitment and retention of young men [1].

Two common recruitment goals are to obtain a sample sufficient to represent the target population and to recruit adequate numbers, with power to test the primary hypothesis [6]. Difficulty recruiting participants can disrupt the study timeline and consequently strain resources and ability of researchers to complete the study as planned within budget and time limits [7]. Strategies traditionally used to recruit young women have included random digit dialing, media and community advertising campaigns or university-based recruitment [8].

Challenges of making initial contact with young women and then maintaining it appear to be associated with age-normative transitional events [5], including commencement of university studies, moving residence, commencing new employment, travelling overseas, and time constraints related to work, study, co-habitation, family and peers. Strategies commonly used to facilitate retention include the use of phone calls, sending out reminder text messages, Email reminders or mail [2,5].

New methods to recruit young women are being explored and include the use of social media such as Facebook and Twitter [9,10]. There is growing evidence on the use of social media for study recruitment [10]. Many social interactions with young women occur via the Internet, therefore social networking sites are being utilised more frequently to recruit participants [4]. The Australian Longitudinal Study of Women’s Health (ALSWH) is now recruiting a new young cohort of females aged 18 to 23 years. Recruitment for this new young cohort is being conducted using the ALSWH website, which includes a link to their Facebook page and Twitter account, and offers incentives to participants [11]. The use of social media in recruitment is somewhat limited in its ability to reach individuals who have little or no access to the Internet, however, having Internet access is very common. The Australian Communications and Media Authority reported that as of June 2013, 10.8 million Australians accessed the Internet once per day, a 7% increase from June 2008 [12]. Also in 2012, a US report confirmed that Internet usage was not restricted to higher socio-economic status with 75% of lower socio-economic individuals using the Internet and 66% of adult Internet users accessing social media sites [10].

In this paper, we examine recruitment and retention across three recent studies in young women. Our aim is to address the practical considerations surrounding recruitment and retention at this life stage and to make recommendations for optimising these aspects within future research.

## Methods

Comparative analysis of the processes used to recruit and retain young women in three research studies conducted at The University of Newcastle, New South Wales, Australia was conducted. Methodological details for each study, participant characteristics and retention rate are outlined in Table 1. Effectiveness of recruitment and retention was compared based on the numbers of women who contacted researchers and expressed initial interest in participation; numbers screened for eligibility; numbers eligible/ineligible for study inclusion and reason for ineligibility; numbers commencing the study; numbers completing the study; and reasons for withdrawing.

**Table 1:** Details of three studies that recruited young women, conducted at the University of Newcastle, Australia

**Study**	**Participants and eligibility criteria**	**Recruitment**	**Retention strategies**
Study one (iron and cognition RCT)	● Women aged 18 to 35 years	Recruitment location and time period:	● Text message reminders for taking capsules
	● BMI 18.5 to 30 kg/m^2^
	● English as first language	● University of Newcastle, Australia from August 2010 to March 2013	● Recommendation to leave container of capsules next to their toothbrush
	● Not diagnosed with iron deficiency within the last 12 months
	● Not currently taking iron supplementation	Recruitment methods:	● Recommendation to use the calendar provided to cross days off after the capsule was taken
	● No chronic medical condition	● Flyers distributed across the campus (notice boards, cafeterias and outside lecture theatres).	● A small container was provided for handbags if remembered later in the day
	● Not taking medication that could potentially interfere with results	● In lectures, using PowerPoint slide	● This information was included in a tips sheet for participants
	● Not donated blood within three months prior to screening	● Word of mouth around campus	● Refer to information sheet provided if common symptoms were present
	● Able to provide blood samples	● Provision of course credit in two University courses (psychology and nursing)	
	● Not pregnant, or planning to become pregnant within the following four months	● Hunter Medical Research Institute (HMRI) research volunteer register	
	● Available for the following four months.	● Community flyers (gyms, technical college campuses and by word-of-mouth).	
		● Emails sent around University mailing lists for each faculty	
		● Advertisement on the University of Newcastle Facebook page.	
Study two (crossover food diary validation study)	● Women aged 18 to 30 years	Recruitment location and time period:	● Email and text message reminders about each data collection session
	● Healthy weight or overweight (BMI 21 to 30 kg/m^2^)	● University of Newcastle, Australia from January to May 2012.	● Thirty dollar reimbursement gift voucher to cover travel and parking costs
	● Access to a computer and a Smartphone with Internet access	Recruitment methods:	
	● Self-reported moderate level of Internet and Smartphone skills	● Advertisements posted on staff and student bulletin boards,	
	● Weight stability over previous three months, and willingness to remain weight stable over the 37-day study period	University website and social networking sites (that is, Facebook and Twitter).	
	● Not currently or planning to become pregnant or currently breastfeeding		
	● Not taking medications that affect weight or appetite		
	● No diagnosed metabolic disorders		
	● Non-smokers		
Study three (cross-sectional online weight management survey)	● Australian women aged 18 to 30 years	Recruitment location and time period:	N/A
		● University of Newcastle in August 2012	
		Recruitment methods:	
		● Advertisements on University website	
		● Social networking sites	
		● Staff and student Email lists	
		● A link to the online survey was provided as part of the advertisement/Email.	
		● ‘Virtual snowballing’ was used to increase the size, and representative nature of the sample, whereby survey respondents were asked to pass on details of the study to others within the target group via Email (that is, Email the survey link to their friends) and/or social networking (for example, share the survey link with their Facebook friends)	
		● Prize draws were used to attract young women, including shopping centre, beauty therapy and cinema vouchers.	

### Study descriptions

Study one was a double-blind, placebo-controlled, randomized trial. The aim was to examine the efficacy of iron supplementation in iron deficient women and its effect on cognitive function. Pre and post assessments involved completing a 50 minute computer-based cognition test, and having a blood sample taken on campus. Iron deficient participants and a proportion of iron sufficient participants continued to the intervention phase where they were given placebo, 60 mg or 80 mg elemental iron for 16 weeks. The target sample was 120 women. Participants received personal feedback regarding their blood test results.

Study two used a randomised crossover design to compare the accuracy and acceptability of a web-based food diary completed via computer, to a food diary accessed via a Smartphone, to a paper-based food diary. Young women (18 to 30 years of age) completed all three food diary modes, over three separate seven-day periods with the order of completion assigned randomly (days 2 to 8, days 16 to 22 and days 30 to 36). On day 1, participants’ resting energy expenditure was measured via indirect calorimetry, as well as their height and body composition. From day 2 to day 8, their physical activity levels were monitored via accelerometry. On days 1, 9, 15, 23, 29 and 37, their weight was measured, and on days 9, 23 and 37, they completed a survey regarding the acceptability of the food diary completed. Participants attended assessment sessions on six occasions over 37 days, during which measures were taken for demographics, weight, height, and body composition. The length of sessions varied from ten minutes to one hour, depending on which assessments were being measured. The target sample was 40 women.

Study three was a brief (approximately 15 minutes) online cross-sectional survey to identify expectations of an eHealth weight management intervention (for example, mode of delivery, content, features). The aims were also to identify barriers to weight loss overall, as well as specific barriers to participating and engaging in an eHealth weight loss intervention. The target sample was at least 100 women.

## Results

### Comparing the effectiveness of recruitment

In study one a total of 155 participants expressed interest in participating from June 2010 to March 2013. Of these, 128 participants met eligibility criteria. The recruitment methods used during the first 12 months were flyers on campus notice boards at the University of Newcastle and the Newcastle Technical and Further Education (TAFE) College, Emails to University staff, promotion in lectures and word-of-mouth. Seventy three participants (57% of eligible participants) were recruited within the initial 12 months. From 2012 to 2013 recruitment was extended to the wider community and included flyers in gyms and day care centres, word-of-mouth, and Facebook messages. Advertising the study in the Research Awareness Exercise Program for two University courses (psychology and nursing) was also included. Participation in a research awareness program enabled students to receive course credit for their participation, with 15 participants (12% of total) recruited via this method. Another approach undertaken from 2012 to 2013 was to extend community-based recruitment and utilise the Hunter Medical Research Institute (HMRI) volunteer register. Questionnaires were sent to 250 females who had placed themselves on the register, resulting in recruitment of 10 participants (8% of total). Repeating community-based methods and refreshing University campus flyers attracted the final 30 participants from 2012 to March 2013.

In study two, 91 individuals expressed interest, of whom 22 participated (24%). For this study, flyers were displayed on University campus noticeboards and Emailed to staff and students. The University’s media unit also advertised it on the University website news, with a link to the story placed on the University’s Facebook site, and details released via Twitter.

In study three, the recruitment methods used generated the required study sample (> 100) one month, the shortest time frame of the three included studies. The study was advertised via the University’s media unit on the University website news, and promoted on the University’s social media sites (Facebook and Twitter). Invitations to participate were also distributed to staff and students across various University Email lists. Participants were encouraged to share the survey link at the end of the study with their friends via social networks or Email. Of the 798 individuals who expressed interest, 751 (94%) were assessed as eligible, and 570 (71.4%) completed the full survey (see Table 2).

**Table 2:** Length of recruitment and participant flow in each of the three included studies

**Contact and screening**	**Study one (RCT) n (%)**	**Study two (crossover validation) n (%)**	**Study three (cross-sectional online survey) n (%)**
Length of recruitment (months)	36	4	1
Expressed interest in participating (n)	155	91	798
Number completing eligibility screening	134 (86)	83 (91)	780 (97)
Number of screened participants who were eligible	128 (95)	22 (26)	751 (96)
Number of eligible participants who completed the study	95 (74)	22 (100)	570 (75)

### Reasons for ineligibility

In study one, of the 134 participants who completed eligibility screening, six participants were not eligible. Of these, one was unable to attend the assessment session at the University, two were male and three had donated blood within three months prior to screening.

Study two screened 83 women, of whom 61 were ineligible. Of these, 22 did not meet body mass index (BMI) criteria, 18 were unable to make the assessment sessions at the University, six did not meet the definition for weight stability in the previous three months, five did not have a Smartphone, five were unwilling to remain weight stable during the study period, two were smokers, two had chronic health issues and one had no access to the Internet.

Study three screened 780 women, of these 29 were deemed ineligible. Four were male, 13 did not meet age criteria and 12 did not live in Australia. As shown in Table 2, 18 of the 798 did not complete the eligibility screening questions.

### Retention strategy effectiveness

Retention strategies used by study one and study two are summarised in Table 1, and include text message and Email reminders. Of the 128 eligible for study one, 95 (74%) provided complete data sets at baseline testing, 32 were subsequently enrolled in the RCT of whom 26 (81%) completed the 16 week intervention and follow-up testing. Of the six participants who commenced but did not complete the intervention, reasons given for withdrawing included unrelated illness (n = 4) or being too busy (n = 2).

Methods used to assist with retention and adherence in study one included optional text message reminders, a paper-based calendar, a small capsule container for carrying supplements in a handbag, and four-weekly symptom check phone calls. These methods are detailed in Table 1. Only 10/32 participants opted to have text message reminders sent to them. All ten of these participants reported that they liked receiving the reminders. All except one participant in study one remained contactable by phone or Email for the entirety of the study. Of the 124 capsules provided to participants, the average number of capsules remaining and returned to researchers was 12 ± 12 (9.6%). Data on the number of capsules missed indicated that receiving a text message reminder made no difference to the number of capsules missed.

All 22 women in study two had completed the study within a six-month recruitment period (January to June 2012) but only 18 completed more than 85% of recording days for each diary and provided enough data to be included in the study analysis.

## Discussion

This paper examined data related to recruitment and retention of young women across three different research study designs in order to address the practical issues related to their participation and representation within research. The results showed that the use of social media, text messages and face-to-face contact were beneficial in the recruitment of young women into nutrition research studies. These results are similar to those of a 2009 Cochrane Review by Mapstone et al. [13] that identified 15 trials evaluating the effectiveness of strategies to improve recruitment in research. They reported that trials using monetary incentives, and treatment information on the consent form demonstrated benefit [13]. Furimsky et al. (2008) examined the challenges of recruiting and retaining youth with mental illness within RCTs and found incentives and flexibility in scheduling useful [14]. Neither of these studies examined the influence of gender on recruitment.

Adamson et al. (2007) scrutinized recruitment strategies used in the Australian Longitudinal Study on Women’s Health (ALSWH). Authors reported on the importance of piloting recruitment strategies and keeping records of recruitment successful processes [15]. They also recommended ensuring contact could be made easily by participants and in 1996 when this study was recruiting this was by providing a free call number to participants and obtaining as many inward phone lines as possible. At this time, when there was limited access to the Internet, phone contact was one of the easiest methods of accessing participants as it provided an instant response.

Each of the three studies examined in this paper placed recruitment information on social networks online and found such strategies particularly useful when recruiting this demographic. A recent study by Fenner et al. also found modern information and communication technologies useful in assisting in engaging young women in health research [4]. Findings demonstrating the utility of recruiting young participants using social media sites, such as Facebook and Twitter are not surprising when you consider that three-quarters of adult Internet users under the age of 25 users have profiles on social media sites [16]. The large number of people connected to the Internet means a large potential pool of participants [17]. Study one had social media added to recruitment methods after an initial 12 months of slow recruitment. Study two used social media from the beginning of recruitment and recruited 22 participants within four months, which was slightly slower than study one, which recruited 31 participants within the same timeframe. Study three recruited 751 participants in one month. It is important to note that both study one and two (crossover validation) had reasonably stringent eligibility criteria and had higher levels of participant burden than study three, therefore making recruitment more difficult. Reasons for ineligibility were recorded for each study, which gave insight about the barriers to successful enrolment into a study, even after individuals have expressed interest in research studies. A barrier is not being able to attend the face-to-face assessment sessions.

Study one also included a mail-out of study information to volunteers who had placed themselves on a research register, in its recruitment methods. Less than 10% of participants were recruited using this method. This recruitment rate was considerably lower than the 40% response rate reported by [17]. Potential reasons for the low number of participants recruited via this method in study one may be the time burden associated with having to attend the University for blood and cognition tests.

Traditional methods such as flyers, PowerPoint slides in lectures, staff and student Email lists were used by the studies. These methods were effective in recruiting the majority of the participants in study one, however, resulted in slow recruitment, and required regular refreshing of flyers on the noticeboards.

Direct comparison of recruitment between the three studies included was not an aim due to differences in the degree of burden associated with participation, and in the difference influence of incentives across the studies. The blood test required for study one was the most significant participant burden of the three studies. Study one also included taking capsules every day for a 16-week period. Study two involved burden associated with completing lengthy food records and having six face-to-face assessments. High participant burden is more likely to be acceptable if the personal benefit of study results is also high. For example, the numbers of iron deficient females volunteering for the RCT early on in recruitment was significantly greater than the previously reported prevalence of iron deficiency in this age group, suggesting that these participants potentially had personal health benefits as a factor motivating participation.

Previous research has shown that when recruiting for an RCT, the use of incentives such as feedback, course credit, money or ‘lottery’ is useful [2]. Various incentives were offered in all studies. Study one offered individual feedback on blood test results and study two offered feedback on participants’ diets.

Study one was advertised to students in psychology and nursing courses that offer course credit for research participation. Both psychology and nursing are large courses at the University of Newcastle (950 and 1,500 students, respectively) with high percentages of females. The ‘value’ of the credit is limited to five (nursing) or ten (psychology) marks out of 100 and it seems this may not be adequate incentive for large numbers of students to participate. In addition, students may have other research studies competing for their interest.

Studies two and three offered vouchers as incentives for participants. The chance to win ten shopping vouchers valued at 150 dollars is likely to have assisted in the recruitment of 400 participants (70%) within the first day of online recruitment into study three. The addition of a monetary or voucher based incentive may have improved recruitment for study one.

The lower burden of the cross-sectional design, not involving blood testing, capsules or extensive food diaries [18] and the short one-off online survey with an incentive of prize draws is very likely to have made study three more appealing to participants. It is possible to recruit a lot of young women quite quickly if incentive prize draws are offered and combined with the convenience on online participation that is one-off. The National Statement on Ethical Conduct in Human Research in Australia approve the reimbursement of participants for costs associated with participation, however, state that it is unacceptable to provide payment to participants that is disproportionate to the time involved or encourages them to take risks [19].

Our findings suggest that text reminders, phone calls and face-to-face contact may improve retention of young women in nutrition research, however further research is required to compare different incentives and contact methods to enable more definitive conclusions. The recruitment of young males into research may also benefit from using the strategies identified and further research in young males is required.

### Recommendations for researchers

A number of key recommendations emerge from our examination of recruitment and retention methods across three studies:

1. Social networking sites should be utilised to distribute or advertise the study.

2. Consider participants’ motivation for participating, such as health benefits or incentives.

3. Use appropriate reimbursement or incentives such as monetary reward relative to the time demand involved, vouchers and course credit that are targeted to the population group.

4. Be flexible regarding testing days and times and provide individual feedback of results where appropriate.

5. Speak with participants in person or on phone calls as soon as possible to build researcher-participant rapport.

6. Use Email, phone calls, text messages and face-to-face contact as much as possible to maintain communication with participants.

## Conclusion

This paper aimed to address the practical considerations surrounding recruitment and retention of young women in research studies. Recruiting young women for intervention trials is challenging. However, strategies such as using technologies (for example, social networking, Email, text messages) already used by young women can facilitate both recruitment and retention. The appropriate use of incentives and Email or phone reminders are important and should be planned from the start in order to optimize retention and study quality, statistical power and research outcomes.

## Abbreviations

RCT: Randomised Controlled Trial; ALSWH: Australian Longitudinal Study of Women’s Health; HMRI: Hunter Medical Research Institute; TAFE: Technical and Further Education; BMI: Body Mass Index.

## Competing interests

The authors declare that they have no competing interests.

## Authors’ contributions

AL made substantial contribution to conception and design; conducted one of the studies analysed and significantly contributed to the preparation and revision of the manuscript. MH conducted two of the studies analysed and was involved in the drafting and critical revision of the manuscript for important intellectual content. AP, KC and CC were involved in the drafting and critical revision of the manuscript for important intellectual content. All authors have contributed and approved the final version of the manuscript.

## Funding

Study one was supported by Meat and Livestock Australia, studies two and three by the Priority Research Centre in Physical Activity and Nutrition. Alecia J Leonard receives a University of Newcastle Scholarship and a top-up scholarship from Meat and Livestock Australia. Clare E Collins is supported by a National Health and Medical Research Council Australian Career Development Fellowship.

## References

[B1] Stoy D Mastorianni A Faden R Federman D Women and Health Research 1999 Washington DC. National Academy of Sciences: Ethical and Legal Issues of Including Women in Clinical Studies, Volume 2 264

[B2] Griffin HJ O’Connor HT Rooney KB Steinbeck KS Effectiveness of strategies for recruiting overweight and obese generation Y women to a clinical weight management trial Asia Pac J Clin Nutr 2013 22 2 235 40 23635367 10.6133/apjcn.2013.22.2.16

[B3] Hure AJ Smith R Collins CE A recruiting failure turned success BMC Health Serv Res 2008 8 64 10.1186/1472-6963-8-64 18366805 PMC2292709

[B4] Fenner Y Garland SM Moore EE Jayasinghe Y Fletcher A Tabrizi SN Gunasekaran B Wark JD Web-based recruiting for health research using a social networking site: an exploratory study J Med Internet Res 2012 14 1 e20 22297093 10.2196/jmir.1978 PMC3374531

[B5] Faden VB Day NL Windle M Windle R Grube JW Molina BSG Pelham WE Gnagy EM Wilson TK Jackson KM Sher KJ Collecting longitudinal data through childhood, adolescence, and young adulthood: methodological challenges Alcohol Clin Exp Res 2004 28 2 330 340 10.1097/01.ALC.0000113411.33088.FE 15112941 PMC2898727

[B6] Hulley S Cimmings S Browner W (Eds): Designing Clinical Research. An Epidemiological Approach. 2nd edition 2001 London: Lippincott Williams and Wilkins

[B7] Ashery R McAuliffe W Implementation issues and techniques in randomised controlled trials of outpatient psychosocial treatments for drug abusers Recruitment of subjects. Am J Drug Alcohol Abuse 1992 18 305 329 10.3109/00952999209026069 1329493

[B8] Morton LM Cahill J Hartge P Reporting participation in epidemiologic studies: a survey of practice Am J Epidemiol 2006 163 3 197 203 16339049 10.1093/aje/kwj036

[B9] Eagan J Health Benefits of Social Media 2013 Health Journey Available from: [http://www.womhealth.org.au/healthy-lifestyle/191-health-benefits-of-social-media]

[B10] Lohse B Facebook is an effective strategy to recruit low-income women to online nutrition education J Nutr Educ Behav 2013 45 1 69 76 10.1016/j.jneb.2012.06.006 23305805

[B11] Women’s Health Australia Australian Longitudinal Study on Women’s Health 2013 [cited 16 December 2013]; Available from: [http://www.alswh.org.au/for-participants/1989-94-cohort]

[B12] Broadcasting A Corporation: annual report 2012 to2013 Australian Broadcasting Corporation Authority Melbourne 2013 2013

[B13] Mapstone J Elbourne D Roberts I Strategies to improve recruitment to research studies Cochrane Database Syst Rev. BMJ Open 2012 2 e000496 doi:10.1136/bmjopen-2011-000496 10.1002/14651858.MR000013.pub3 17443634

[B14] Furimsky I Cheung AH Dewa CS Zipursky RB Strategies to enhance patient recruitment and retention in research involving patients with a first episode of mental illness Contemp Clin Trials 2008 29 6 862 866 10.1016/j.cct.2008.07.005 18721902

[B15] Adamson L Young A Byles EJ Recruiting for a longitudinal study: who to choose, how to choose and how to enhance participation Int J Mult Res Approaches 2007 1 126 136 10.5172/mra.455.1.2.126

[B16] Correa T Hinsley AW de Zuniga HG Who interacts on the Web?: the intersection of users’ personality and social media use Comput Hum Behav 2010 26 2 247 253

[B17] Hoonakker P Carayon P Questionnaire survey nonresponse: a comparison of postal mail and Internet surveys Int J Hum-Comput Interact 2009 25 5 348 373 10.1080/10447310902864951

[B18] Patel M Doku V Tennakoon L Challenges in recruitment of research participants J Contin Prof Dev 2003 9 229 238

[B19] National Health and Medical Research Council, Australian Research Council, Australian Vice-Chancellors’ Committee Committee: National Statement on Ethical Conduct in Human Research (Updated May 2013) 2007 Commonwealth of Australia Canberra, Australia: Australian Government

